# External Replication of Urinary Bladder Cancer Prognostic Polymorphisms in the UK Biobank

**DOI:** 10.3389/fonc.2019.01082

**Published:** 2019-10-18

**Authors:** Nadezda Lipunova, Anke Wesselius, Kar K. Cheng, Frederik J. van Schooten, Jean-Baptiste Cazier, Richard T. Bryan, Maurice P. Zeegers

**Affiliations:** ^1^Institute of Cancer and Genomic Sciences, University of Birmingham, Birmingham, United Kingdom; ^2^Department of Complex Genetics, Maastricht University, Maastricht, Netherlands; ^3^Centre for Computational Biology, University of Birmingham, Birmingham, United Kingdom; ^4^Institute for Applied Health Research, University of Birmingham, Birmingham, United Kingdom; ^5^Department of Pharmacology and Toxicology, Maastricht University, Maastricht, Netherlands

**Keywords:** bladder cancer, SNP, replication, UK Biobank, prognosis

## Abstract

**Introduction:** Multiple studies have reported genetic associations with prognostic outcomes of urinary bladder cancer. However, the lack of replication of these associations prohibits establishing further evidence-based research directions. Moreover, there is a lack of independent bladder cancer patient samples that contain prognostic measures, making genetic replication analyses even more challenging.

**Materials and Methods:** We have identified 1,534 eligible patients and used data on Hospital Episode Statistics in the UK Biobank to model variables of otherwise non-collected events on bladder cancer recurrence and progression. Data on survival was extracted from the Death Registry. We have used SNPTEST software to replicate previously reported genetic associations with bladder cancer recurrence (*N* = 69), progression (*N* = 23), survival (*N* = 53), and age at the time of diagnosis (*N* = 20).

**Results:** Using our algorithm, we have identified 618 recurrence and 58 UBC progression events. In total, there were 209 deaths (106 UBC-specific). In replication analyses, eight SNPs have reached nominal statistical significance (*p* < 0.05). Rs2042329 (*CWC27*) for UBC recurrence; rs804256, rs4639, and rs804276 (in/close to *NEIL2*) for NMIBC recurrence; rs2293347 (*EGFR*) for UBC OS; rs3756712 (*PDCD6)* for NMIBC OS; rs2344673 (*RGS5)* for MIBC OS, and rs2297518 (*NOS2*) for UBC progression. However, none have remained significant after adjustments for multiple comparisons.

**Discussion:** External replication in genetic epidemiology is an essential step to identify credible findings. In our study, we identify potential genetic targets of higher interest for UBC prognosis. In addition, we propose an algorithm for identifying UBC recurrence and progression using routinely-collected data on patient interventions.

## Introduction

Urinary bladder cancer (UBC) is a disease of great burden; yet the diagnosis, clinical management, and patient survivorship has changed little over the last few decades (1, 2). Genetic studies may provide important clues on biological pathways underlying the development of UBC. Importantly, advances in understanding what drives a favorable UBC prognosis could aid in predicting patient outcomes. As a result, and informed and timely patient stratification would allow an individually-tailored cancer management plan, which is likely to better reflect patient needs than current group-level recommendations (3).

Multiple genetic associations with UBC prognostic outcomes (e.g., survival, recurrence) have been reported in the literature (Lipunova et al., under review). However, the number of potential genetic clues far exceeds the available resources for clinical and functional investigation. As such, the scientific community must take an approach of targeting most-promising associations first.

There are multiple ways to define clinical relevance of a genetic variant, including external replication to reduce the chance of false-positives (4, 5). However, replication of genetic associations includes many hurdles, such as a lack of independent participant cohorts with adequate sample sizes. Moreover, focus on a sub-phenotype (e.g., recurrence) makes it even more difficult due to required additional sources of data (e.g., hospital records).

Increased availability of population-based electronic health records can help to alleviate the burden of investigating diseases for which adequate sample sizes are difficult to acquire. UK Biobank is the largest population-based cohort in the United Kingdom and serves as a powerful resource for investigating genetic associations (6) and has not yet been widely used for investigating UBC. The presence of Hospital Episode Statistics (HES) in the UK Biobank offers an unprecedented opportunity to use these data to identify UBC recurrence and progression events, that are not a part of the usually-collected information.

In the current study, we have aimed to identify UBC patients in the UK Biobank and use HES statistics to construct prognostic events. We have further used this data to externally replicate previously reported genetic associations on UBC survival, recurrence, and age at the time of diagnosis.

## Materials and Methods

### SNP Selection

**Figure 1:**
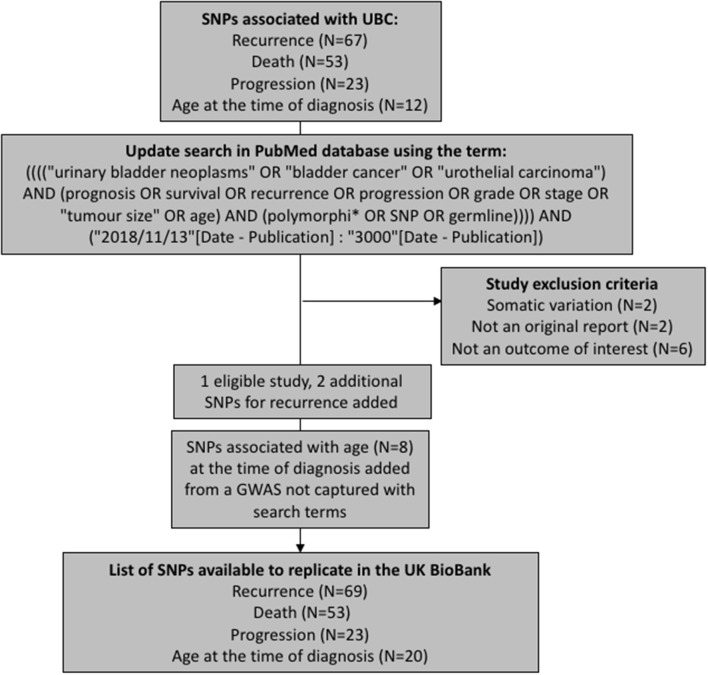
Selection process of the SNPs used in replication analyses.

We have aimed to replicate all SNPs that have been previously associated with UBC recurrence, progression, death (overall or cancer-specific), and age at the time of diagnosis. The polymorphisms were extracted from a recent review on prognostic UBC outcomes (Lipunova et al., under review). To capture any associations reported since the review, we have updated the list of SNPs by querying PubMed database for new articles using identical search terms to those used in the review (Figure 1). The search was limited to articles published in English language between 13th November 2018 and 19th February 2019. Eleven papers were identified in total, with one study being eligible for inclusion (7). Additionally, we have included associations for age at the time of diagnosis from a genome-wide association study (GWAS) previously carried out in the Bladder Cancer Prognosis Programme (BCPP) (8).

After removing duplicate entries, there were 69 SNPs to test for recurrence, 53 for survival, 20 for age, and 23 for progression (Supplementary Tables 1–4).

### Study Population

UK Biobank is a population-based cohort in the UK, having collected genetic and clinical data on over 500,000 participants, aged 40–69 at the time of recruitment in 2006–2010. The design, data collection and processing are described in detail elsewhere (6, 9).

Our analysis was restricted to UBC patients (corresponding International Classification of Diseases (ICD) codes of C67.0, C67.1, C67.2, C67.3, C67.4, C67.5, C67.6, C67.7, C67.8, C67.9, D09.0 (ICD10) and 1880, 1882, 1884, 1886, 1888, 1889, 2337 (ICD9). To prevent bias from analyzing heterogeneous molecular UBC subtypes, histology was limited to the following ICD-O (ICD Oncology) codes: 8000 (Neoplasm), 8001 (Tumor cells), 8010 (Carcinoma), 8020 (Carcinoma, undifferentiated), 8050 (Papillary carcinoma), 8120 (Transitional cell carcinoma), and 8130 (Papillary transitional cell carcinoma).

HES contains admitted in-patient data starting with 1997 (10) and includes data on patients both under National Health Service (NHS) and private care. HES data is provided to the UK Biobank on an annual basis, covering the past financial year (starting 1st April of each year). In our analyses, the follow-up covers all in-hospital interventions registered until March 31st, 2017. Operative procedures use OPCS4 (Office of Population, Censuses and Surveys: Classification of Interventions and Procedures, Version 4) coding system.

In total, there were 1,534 UBC patients with clinical and genetic data available for analysis.

### Outcomes

#### Age

Age at the time of diagnosis was modeled both as a continuous and categorical variable.

To replicate previous associations as accurately as possible, we have dichotomised age variables using the cut-off points reported in the original research articles (≥/ <50, 55, 60, 65, and 70 years, Supplementary Table 3).

#### Death

Death was modeled as an overall (death vs. no death) or a UBC-specific event (death vs. no death, when primary cause of death was assigned C67-(ICD10) or 188-related (ICD9) codes).

#### Recurrence

The events of bladder recurrence and progression are not part of the routinely collected data in the Cancer Registry, or other national/regional datasets. However, the HES in the UK Biobank make it possible to identify a fraction of these events using proxy data.

**Figure 2:**
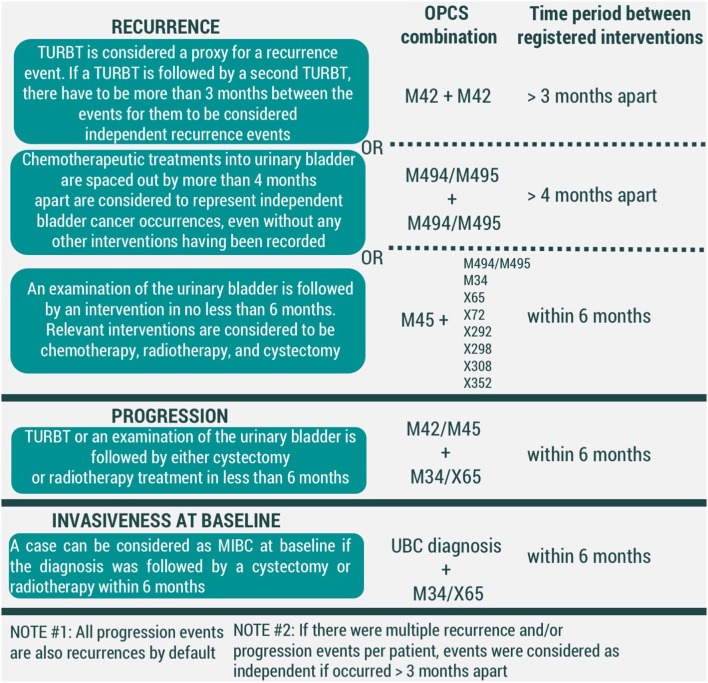
Conditions for modeled events of UBC recurrence, progression, and invasiveness at baseline (MIBC-muscle-invasive bladder cancer, TURBT-transurethral resection of bladder tumor). All codes correspond to OPCS4 classification.

For recurrence, we have considered three conditions to be representative of an event (Figure 2). First, a transurethral resection of a bladder tumor (TURBT) (OPCS4 code M42) is regarded to be enough to signify a UBC event. Secondly, a time gap of longer than 4 months between chemotherapeutic treatments into urinary bladder (OPCS4 codes M494/M495) was considered to be substantial to correspond to two different events. Thirdly, we have assumed a UBC diagnosis if an examination of the urinary bladder (OPCS4 code M45) was led by an intervention within 6 months. Relevant interventions were chemotherapeutic treatments into urinary bladder, cystectomy, radiotherapy, and chemotherapy (corresponding to OPCS4 codes of M494/M495, M34, X65, X72, X292, X298, X308, X352, respectively). Currently presented list of chemotherapy-related OPCS4 is not exhaustive, but rather based on interventions observed in our data. Further development of the algorithm is likely to adjust the list as needed.

#### Progression

In our framework, all events of progression are recurrences by default. However, we have considered adding additional criteria would allow distinguishing which recurrences were also representative of UBC progression. We have considered an event of UBC progression to have taken place if either a TURBT (OPCS4 code M42) or examination of the urinary bladder (OPCS4 code M45) was followed by interventions of cystectomy (OPCS4 code M34) and/or radiotherapy (OPCS4 code X65) within 6 months (Figure 2).

To prevent registration duplicates, two recurrence and/or progression events were considered independent of one another if time in between the records was >3 months.

#### Invasiveness at the Time of Diagnosis

Finally, UBC clinical management is heavily dependent on its' invasiveness at the initial diagnosis. A UBC diagnosis that was followed by either cystectomy or radiotherapy was considered to represent a muscle-invasive bladder cancer (MIBC), while the remaining diagnoses are held to be non-muscle-invasive bladder cancer (NMIBC) cases (Figure 2).

### Ethics and Consent

All UK Biobank participants have provided informed consent. Current research has been conducted using the UK Biobank Resource under Application Number 42772.

### Genotype Data Quality Control (QC) and Imputation

Detailed procedures on QC and imputation in the UK Biobank are described elsewhere (9).

To verify the high quality of all tested SNPs, we have extracted imputation accuracy measures (INFO scores) and MAF (minor allele frequencies) (Supplementary Table 5). INFO scores are computed to estimate the level certainty of imputed SNPs. The value ranges from 0 to 1, with estimates close to 1 representing SNPs imputed with high accuracy (11).

To avoid population stratification bias, we have restricted our sample to a homogenous group of White British participants, as previously identified by the UK Biobank team (9).

### Statistical Analysis

To test for an association between selected SNPs and UBC recurrence, progression, death, and age, we have utilized SNPTEST (https://mathgen.stats.ox.ac.uk/genetics_software/snptest/snptest.html). To estimate Linkage disequilibrium (LD), an online tool was used (https://ldlink.nci.nih.gov/). LD defines the correlation between alleles in a given population. Due to some SNPs being in high LD, it might be difficult to establish which allele is representing the cause, as they are often inherited together. At the same time, linkage equilibrium suggests alleles are inherited independent of one another. Logistic regression using allele dosages was applied to estimate odds ratios (OR) and corresponding confidence intervals (CI) for death, recurrence, progression, and categorical age events; while linear regression was used to estimate the effect of age as a continuous variable. All associations were tested under additive model of inheritance and adjusted for participant sex. To reduce multiple testing, analyses were ran for the outcome that resembled the originally-reported association most closely (e.g., if a variant has been associated with NMIBC recurrence, we have only tested NMIBC patients instead of the whole UBC sample). To better estimate the strength of evidence for replication results, we additionally included calculation of the Bayes Factor (BF). In simple terms, BF can be considered as a ratio of probabilities for two competing hypotheses (for example, the probability of a SNP being associated with an outcome vs. the SNP not influencing the outcome). The ratio provides an estimate that shows the extent of one hypothesis being more (or less) likely than the alternative one. In contrast, the generically-used frequentist approach (resulting in a *p*-value) evaluates the probability of data under a specific hypothesis, which alone does not provide indication of the association strength.

Variants in the replication were considered promising if the nominal statistical significance (*p*-value) has reached <0.05. Bonferroni adjustment per each outcome for multiple comparisons resulted in statistical significance level (*p*-value) to be 0.0007 for recurrence (α = 0.05/69), 0.002 for progression (α = 0.05/23), 0.0009 for survival (α = 0.05/53), and 0.0025 for age (α = 0.05/20).

## Results

In total, 1,534 UBC patients were available for replication analyses of prognostic events (Table 1). Mean age of UBC patients was 61 years, and most were males (78%). Using our algorithm on HES data, we could identify UBC invasiveness at baseline, recurrent, and progressive events for UBC patients in the UK Biobank cohort. Majority of UBC cases were NMIBC (93%). Death was recorded for 209 (13.6%) patients, out of which 106 were UBC-specific. In addition, we estimate 618 patients (40%) have experienced a recurrence, and 58 (3.8%) have had a UBC progression.

**Table 1:** Descriptive characteristics of the UBC patients from the UK Biobank.

	***N***	*p*-Value^*^
**Sex**	**1,534**	**<0.001**
Males (%)	1,197 (78.0)	
Females (%)	337 (22.0)	
**Age [Mean (SD)]**	61.3 (9.0)	
**Death**	**1,534**	**<0.001**
No (%)	1,325 (86.4)	
Yes (%)	209 (13.6)	
**UBC-specific death**	**1,534**	**<0.001**
No (%)	1,428 (93.1)	
Yes (%)	106 (6.9)	
**Recurrence**	**1,534**	**<0.001**
No (%)	916 (59.7)	
Yes (%)	618 (40.3)	
**Progression**	**1,534**	**<0.001**
No (%)	1,476 (96.2)	
Yes (%)	58 (3.8)	
**NMIBC at baseline**	**1,534**	**<0.001**
No (%)	114 (7.4)	
Yes (%)	1,420 (92.6)	

* *Chi-square test for group independence*.

*NMIBC, non-muscle-invasive bladder cancer; SD, standard deviation; UBC, urinary bladder cancer*.

In the replication analyses, eight SNPs have reached a *p*-value of < 0.05 (Table 2). However, none of the variants remained significant after applying Bonferroni-corrections for multiple comparisons (corrected for each tested outcome).

**Table 2:** Replication results that have reached p < 0.05 in the UK Biobank cohort.

**Outcome**	**rsID**	**Locus**	**REF**	**EFF**	**info value**	**MAF, % (all)**	***N* (total) (AA/AB/BB)**	**MAF, % (cases)**	***N* (cases) (AA/AB/BB)**	**MAF, % (controls)**	***N* (controls) (AA/AB/BB)**	**OR**	**(95% CI)**	***P*-value**	**log10(BF)**	**Annotated gene**
UBC recurrence	rs2042329	5q12.3	T	G	1	41	1,534 (255/739/540)	37	618 (88/284/246)	43	916 (167/455/294)	1.26	(1.10; 1.48)	0.001	1.56	CWC27
NMIBC recurrence	rs804256	8p23.1	T	C	0.99	34	1,420 (634.7/604.96/180.4)	37	607 (254.1/261.2/91.7)	32	813 (380.6/343.8/88.6)	1.23	(1.05; 1.43)	0.012	0.74	NEIL2
MIBC overall survival	rs2344673	1q23.3	G	A	1	12	123 (94/29/0)	4	26 (24/2/0)	14	97 (70/27/0)	0.22	(0.05; 0.98)	0.019	0.10	RGS5
NMIBC overall survival	rs3756712	5p15.33	A	C	0.99	38	1,420 (200.4/667.6/552.0)	33	184 (16.9/85.9/81.2)	38	1,236 (183.5/581.7/470.8)	1.29	(1.02; 1.63)	0.03	0.49	PDCD6
NMIBC recurrence	rs4639	8p23.1	A	G	0.99	43	1,420 (472.7/672.9/274.4)	46	607 (182.2/297.0/127.8)	41	813 (290.5/375.8/146.6)	1.20	(1.03; 1.39)	0.02	0.56	NEIL2
NMIBC recurrence	rs804276	8p23.1	G	A	0.99	41	1,420 (496.9/670.1/253.0)	44	607 (200.0/284.5/122.5)	40	813 (296.9/385.6/130.5)	1.17	(1.01; 1.36)	0.04	0.34	–
UBC overall survival	rs2293347	7p11.2	C	T	0.99	11	1,534 (1215.6/303.5/14.9)	8	209 (176.4/31.6/1.0)	11	1,325 (1,039.2/271.9/13.9)	0.69	(0.47; 0.99)	0.04	0.35	EGFR
UBC progression	rs2297518	17q11.2	G	A	1	19	1,534 (999/475/60)	12	58 (45/12/1)	20	1,476 (954/463/59)	0.56	(0.32; 0.99)	0.03	0.26	NOS2

*BP, base pair; BF, Bayes' Factor; CI, confidence interval; EFF, effect allele; MAF, minor allele frequency; MIBC, muscle-invasive bladder cancer; NMIBC, non-muscle-invasive bladder cancer; OR, odds ratio; REF, reference allele; rsID, polymorphism ID; UBC, urinary bladder cancer*.

### Recurrence

Four of these SNPs were associated with bladder cancer recurrence. Rs2042329 (*CWC27*) was linked to an increased risk of UBC recurrence (OR = 1.26, 95% CI: 1.10; 1.48); while rs804256, rs4639, and rs804276, all located in/close to *NEIL2* were associated with NMIBC-only recurrence (OR = 1.23, 95% CI: 1.05–1.43; OR = 1.20, 95%CI: 1.03–1.39; OR = 1.17, 95% CI: 1.01–1.36, respectively). All SNPs that were associated with recurrence showed consistent direction, but were more modest in comparison to the original studies [HR = 1.54 (1.10–2.16) for rs2042329 (12), HR = 4.58 (2.61–8.02) for rs804256 (13), HR = 2.60 (1.68–4.03) for rs4639 (13), and HR = 2.71 (1.75–4.20) for rs804276 (13)].

Although SNPs rs804256, rs4639, and rs804276 all map to the same locus, LD values imply they are independent results (*R*^2^ for rs804276 and rs804256 = 0.09; *R*^2^ for rs804276 and rs4639 = 0.43; *R*^2^ for rs4639 and rs804256 = 0.38).

### Death

Three SNPs [rs2344673 (*RGS5*), rs3756712 (*PDCD6*), and rs2293347 (*EGFR*)] were associated with events of bladder cancer death, albeit in different subgroups. Rs2293347 (*EGFR*) was associated with lower death rates among all UBC patients (OR = 0.69, 95% CI: 0.47–0.99), rs3756712 (*PDCD6)* was significant for NMIBC patients (OR = 1.29, 95% CI: 1.02–1.63), and rs2344673 (*RGS5*) showed reduced rate of death among MIBC cases (OR = 0.22, 95% CI: 0.05–0.98).

In comparison to the original study, replicated SNPs in *PDCD6* showed effect in the same direction, but had a reduced estimate [HR = 5.11 (1.43–18.22) (14)].

However, inconsistency in direction of the effect was observed for SNPs in *EGFR* and *RGS5* [HR = 1.5 (1.0–2.3) for rs2293347 (15) and HR = 1.55 (1.15–2.11) for rs2344673 (16)].

### Progression

Carrying a minor allele of rs2297518 in *NOS2* corresponded to a lower chance of UBC progression (OR = 0.56, 95% CI: 0.32–0.99). In the original study, rs2297518 was also associated with a lower risk of progression [HR = 0.21 (0.05–0.87) (17)].

Bayes factor was highest for the variant associated with NMIBC recurrence in *CWC27*, reaching log10(BF) = 1.56. For all remaining SNPs, Bayes statistic indicates replication sample was low-powered (18), with log10(BF) ranging between 0 and 1.

## Discussion

In the current study, we describe an external replication of previously reported genetic associations for UBC recurrence, progression, death, and age at the time of diagnosis using HES data available the UK Biobank.

The aim of our study is 2-fold. Firstly, mining routinely-collected data for identifying complex phenotypes is inevitable to become a common practice. In the light of current needs, we propose an algorithm that identifies UBC recurrences and progression events via recorded interventions in a hospital setting. Current approach uses OPCS4 classification system, but we are confident applied assumptions can be translated to other globally-used systems (e.g., International Classification of Health Interventions, ICHI). We acknowledge identified prognostic events make up only a fraction of the true event volume, and are likely to be an underestimate. The extent of the underestimation requires testing the algorithm in an external cohort and is a necessary subsequent step in refining the currently-described approach. The level of underestimation is likely to vary for differed outcomes, as some events are arguably easier to identify (e.g., recurrence), while progression requires more detailed data and is subject to a higher level of underrepresentation. However, we saw an overestimation resulting in a greater rate of error and data misrepresentation. Moreover, inclusion of other clinically-relevant characteristics (tumor stage, grade) would increase the accuracy of modeled prognostic events. The provisioned release of such data in the UK Biobank (https://biobank.ctsu.ox.ac.uk/crystal/exinfo.cgi?src=future_timelines) will provide further opportunities of updating the algorithm. Naturally, our proposed approach and assumptions are subjective by nature and we encourage the expert field to contribute ideas to make the assumptions more accurate.

Secondly, an external replication of genetic associations is a rare endeavor. Unfortunately, as simply put by Kraft et al. (4), “*Genetic epidemiology learned the importance of replication the hard way*.” External validation studies perform at much lower rates, which underscores the significance of such efforts (5). Most genetic studies are still exploratory in nature, and false-positive results are inevitable. By prioritizing evidence-based targets, more resources can be allocated toward investigating variants with better promise of true impact on human health.

For UBC recurrence, the strongest result was mapped to *CWC27*. Previous study reported rs2042329 to correspond to higher expression of *CWC27* in bladder cancer cells (12). Additional functional analyses showed *CWC27* might affect bladder carcinogenesis via apoptosis. Interestingly, the original finding was made for Chinese patients, and authors failed to replicate the significance of rs2042329 on bladder cancer risk among Europeans (12). However, it is unknown if the lack of effect was also present for recurrence.

Additionally, it is surprising to see three SNPs in *NEIL2* being significant for NMIBC recurrence, especially keeping in mind the low likelihood of successful replication. Despite the high number of SNPs, strength of evidence for these associations is low, as reflected in Bayes Factor. Nonetheless, they might be promising targets in future replications. *NEIL2* is involved in DNA repair mechanisms, and research suggest it influences malignancies beyond bladder cancer. Alterations in normal *NEIL2* activity most likely result in accumulated oxidative damage, as elegantly presented by Benitez-Buelga et al. (19).

For UBC progression, the replicated variant maps to *NOS2*. The gene has been specifically linked to progression of various cancers (20, 21). It seems *NOS2* affects multiple oncogenic pathways that simultaneously affect tumor proliferation, angiogenesis, chemoresistance, and cell migration (20, 21).

As for UBC survival, three replicated SNPs are located in *RGS5, PCDC6*, and *EGFR*. Interestingly, a previous independent replication of SNPs associated with UBC prognosis has also successfully validated a variant in *RGS5* (rs12035879) for overall survival (OS) of MIBC cases (22). Comparison of two external replications offers potential insights—for example, the rs11585883 did not replicate in our study; however, another SNP in *RGS5* was successful, and associated with the same outcome (MIBC OS). These findings may be seen as cumulative toward the involvement of *RGS5* in cancer survival, even if specific SNPs are yet to be identified. We have checked if previously and current replicated *RGS5* SNPs are in LD, and they seem to represent independent signals in the gene (*R*^2^ = 0.03 for rs12035879 and rs2344673 among Europeans). One major weakness of the replicated rs2344673 in our study is small sample size (29 cases and 109 controls). A *post-hoc* analysis on the overall survival of the whole sample, regardless of UBC invasiveness (209 cases and 1,325 controls) was not significant (data not shown). *RGS5* may not be relevant for all UBC patients, or might reflect power issues, which highlights further investigation being essential.

Remaining two genes implicated in UBC and NMIBC survival, namely *EGFR* and *PDCD6*, are both well-known cancer genes (14, 23). *PDCD6* seems to be heavily involved in apoptosis (14); however, the exact role of *PDCD6* is contrasting between various cancers (24), and further molecular research will help making evidence-based interpretations.

A replicated SNP (rs2293347) in *EGFR* has also previously corresponded to a protective effect on survival of lung cancer patients (25). The effect may be due to higher responsiveness to chemotherapy (26), which is a worthwhile investigation in future analyses.

Or study is subject to limitations, with one of the largest drawbacks being the difference between founders' and replication cohorts. A lot of studies have investigated populations of non-European ancestry, and it is possible we are not able to observe a true effect due to differences in LD of candidate SNPs in different samples. At the same time, the most reliable replication in our study was rs2042329, first reported in a Chinese population (12).

None of our replicated SNPs have passed the Bonferroni-corrected statistical significance level, suggesting some promising SNPs may have been identified by chance. Furthermore, current analyses have only focused on estimating the overall risk of a prognostic event, without considering the relevance of elapsed time to event. We see such and other more sophisticated analyses as a further direction in utilizing the described approach.

We were also unable to reliably estimate assigned treatment for UBC patients in the UK Biobank cohort, which would unquestionably confer to a more precise replication analysis. However, as the detail of released HES is increasing, we do not see this data out of reach and likely to include in future algorithm updates.

Finally, some replicated SNPs showed a conflicting direction of effect when compared to the original studies. These issues are likely to be clarified once more studies can confirm the overall association and establish the effect specifics.

To summarize, we have carried out an external replication of previously reported SNPs for UBC recurrence, progression, death and age using a novel approach of identifying clinically-relevant outcomes using HES data. Our analysis suggests specific targets, namely *CWC27, NEIL2, PDCD6, EGFR*, and *NOS2*, might be prioritized in efforts to further study the role of genetics in UBC prognosis. We are cautious about our findings, as there is no one metric or design to provide unquestionable evidence; instead, it should be viewed as one of the studies in a long line of accelerating research on UBC.

## Data Availability Statement

The raw data supporting the conclusions of this manuscript will be made available by the authors, without undue reservation, to any qualified researcher.

## Ethics Statement

The studies involving human participants were reviewed and approved by NHS National Research Ethics Service North West (11/NW/0382). The patients/participants provided their written informed consent to participate in this study.

## Author Contributions

NL designed the study, organized the data, performed statistical analyses, and wrote the first draft of the manuscript. All authors contributed to the manuscript and study design revision, read, and approved the submitted version.

### Conflict of Interest

The authors declare that the research was conducted in the absence of any commercial or financial relationships that could be construed as a potential conflict of interest.
