# Supplementary material for: External Replication of Urinary Bladder Cancer Prognostic Polymorphisms in the UK Biobank
Source: Front Oncol. 2019 Oct 18;9:1082. doi: 10.3389/fonc.2019.01082 (PMC6813571; doi:10.3389/fonc.2019.01082)
Supplement: Supplementary file 1 [file Data_Sheet_1.PDF]

Supplementary Table 1. Previously reported polymorphisms in association with bladder cancer recurrence.

Outcome	SNP	Locus	Gene	EA	RA	EAF*	Discovery population	References
High-risk NMIBC Recurrence (BCG-treated)	rs2070744	7q36.1	NOS3	T	C	0.77	European (Sweden)	Ryk et al.[1]
High-risk NMIBC Recurrence (BCG-treated)	rs1799983	7q36.1	NOS3	G	T	0.82	European (Sweden)	Ryk et al.[1]
Low-risk NMIBC recurrence	rs744154	16p13.12	ERCC4	C	A	0.22	Chinese	Wang et al. [2]
Low-risk NMIBC recurrence	rs798766	4p16.3	TACC3/FGFR3	T	C	0.24	European (multiple)	Kiemeney et al. [3]
MIBC Recurrence	rs4957014	5p15.33	PDCD6	G	T	0.65	Chinese	Zhou et al. [4]
MIBC recurrence	rs4758680	12q24.31	IL31	A	C	0.29	Chinese	Li et al. [5]
NMIBC and UBC Recurrence	rs2505568	10p11.21 (corrected from the originally reported) 7q22.3	NAMPTP1 (corrected from originally reported NAMPT)	A	T	0.57	Chinese	Zhang et al. [6]
NMIBC Recurrence	rs2910164	5q33.3	MIR146A	C	G	0.28 (ExAC)	Chinese	Wang et al. [7]
NMIBC Recurrence	rs511918	1q25.3	RGS16	T	G	0.47	Caucasian (Northern American)	Lee et al. [8]
NMIBC Recurrence	rs16829458	1q31.2	RGS2	A	G	0.12	Caucasian (Northern American)	Lee et al. [8]
NMIBC Recurrence	rs3795617	1q31.2	RGS13	A	G	0.38	Caucasian (Northern American)	Lee et al. [8]
NMIBC Recurrence	rs11199005	10q26.11	RGS10	A	G	0.35	Caucasian (Northern American)	Lee et al. [8]
NMIBC Recurrence	rs1323291	1q31.2	RGS1	C	A	0.15	Caucasian (Northern American)	Lee et al. [8]
NMIBC Recurrence	rs5742714	12q23.2	IGF1	C	G	0.9	Caucasian (Northern American)	Andrew et al. [9]
NMIBC Recurrence	rs2238151	12q24.12	ALDH2	C	T	0.7	Caucasian (Northern American)	Andrew et al. [9]
NMIBC Recurrence	rs2169830	15q14	THBS1	G	A	0.42	Chinese	Yang et al. [10]
NMIBC recurrence (<64 year-old group)	rs1050450	3p21.31	GPX1	T	C	0.22	Caucasian (Northern American)	Zhao et al. [11]
NMIBC recurrence (BCG-treated)	rs16260	16q22.1	CDH1	A	C	0.24	Caucasian (Northern American)	Lin et al. [12]
NMIBC recurrence (BCG-treated)	rs4645978	1p36.21	CASP9	G	A	0.42	Indian	Gangwar et al. [13]
NMIBC Recurrence (BCG-treated)	rs7003908	8q11.21	PRKDC	G	T	0.33	Indian	Gangwar et al. [14]
NMIBC Recurrence (BCG-treated)	rs9904341	17q25.3	BIRC5	C	G	0.39	Indian	Jaiswal et al. [15]
NMIBC Recurrence (BCG-treated)	rs804267	8p23.1	NEIL2	C	T	0.31	Caucasian (Northern American)	Wei et al. [16]
NMIBC Recurrence (BCG-treated)	rs8191604	8p23.1	NEIL2	C	A	0.18	Caucasian (Northern American)	Wei et al. [16]
NMIBC recurrence (BCG-treated)	rs17235409	2q35	SLC11A1	A	G	0.07	Caucasian (Canadian), Chinese	Decobert et al. [17], Chiong et al. [18]
NMIBC recurrence (BCG-treated)	rs1799782	19q13.2	XRCC1	T	C	0.12	Indian	Mittal et al. [19]
NMIBC recurrence (BCG-treated)	rs2228526	10q11.23	ERCC6	C	G	0.18	Caucasian (Northern American)	Gu et al. [20]
NMIBC recurrence (BCG-treated)	rs25487	19q13.2	XRCC1	A	G	0.26	Indian	Mittal et al. [19]

NMIBC recurrence (BCG-treated)	rs1799793	19q13.3	ERCC2	A	G	0.19	Indian	Gangawar et al. [21]
NMIBC recurrence (BCG-treated)	rs2430561	12q15	IFN-G	A	T	0.28	Indian	Ahirwar et al. [22]
NMIBC Recurrence (BCG-treated)	rs2228001	3p25.1	XPC	C	A	0.32	Indian	Gangwar et al. [23]
NMIBC Recurrence (BCG-treated)	rs6463089	7p14.1	GLI3	A	G	0.09	Caucasians (Northern American) + European (Spain) for replication	Chen et al. [24]
NMIBC Recurrence (BCG-treated)	rs3801192	7p14.1	GLI3	A	G	0.07	Caucasians (Northern American) + European (Spain) for replication	Chen et al. [24]
NMIBC Recurrence (BCG-treated)	rs1233560	7q36.3	SHH	G	A	0.46	Caucasians (Northern American) + European (Spain) for replication	Chen et al. [24]
NMIBC Recurrence (BCG-treated)	rs11685068	2q14.2	GLI2	A	G	0.06	Caucasians (Northern American) + European (Spain) for replication	Chen et al. [24]
NMIBC Recurrence (BCG-treated)	rs243865	16q12.2	MMP2	C	T	0.86	Indian	Srivastava et al. [25]
NMIBC Recurrence (BCG-treated)	rs804276	8p23.1	NEIL2	G	A	0.62	Caucasian (Northern American)	Wei et al. [16]
NMIBC Recurrence (BCG-treated)	rs4639	8p23.1	NEIL2	G	A	0.47	Caucasian (Northern American)	Wei et al. [16]
NMIBC Recurrence (BCG-treated)	rs2173962	21q22.11	SOD1	G	A	0.06	Caucasian (Northern American)	Wei et al. [16]
NMIBC Recurrence (BCG-treated)	rs187238	11q23.1	IL18	C	G	0.79	Indian	Jaiswal et al. [26]
NMIBC Recurrence (BCG-treated)	rs1695	11q13.2	GSTP1	G	A	0.35	Chinese	Deng et al. [27]
NMIBC Recurrence (BCG-treated)	rs4925	10q25.1	GSTO1	A	C	0.1	Chinese	Deng et al. [27]
NMIBC Recurrence (BCG-treated)	rs3138056	14q13.2	NFKBIA	T?	C?	0.38 (T)	Northern American	Williams et al. [28]
NMIBC Recurrence (BCG-treated)	rs1544410	12q13.11	VDR	A	G	0.3	Chinese	Wang et al. [29]
NMIBC Recurrence (BCG-treated), NMIBC Recurrence	rs2279744	12q15	MDM2	G	T	0.37	Indian, Chinese	Gangwar et al. [30], Xie et al. [31]
NMIBC recurrence (BCG-treated), NMIBC recurrence (maintenance BCG-treated)	rs1800795	7p15.3	IL6	C	G	0.14	Indian, Caucasian (White)	Ahirwar et al. [32], Leibovici et al. [33]
NMIBC recurrence (BCG-treated), NMIBC Recurrence (TUR- and BCG-treated)	rs1799964	6p21.33	TNFA	C	T	0.22	Indian, European (Southern Portugal)	Ahirwar et al. [34], Lima et al. [35]
NMIBC Recurrence (Epirubicin-treated)	rs915927	19q13.2	XRCC1	G	A	0.32	Chinese	Li et al. [36]
NMIBC Recurrence (Epirubicin-treated)	rs2854501	19q13.2	XRCC1	T	C	0.18	Chinese	Li et al. [36]
NMIBC recurrence (non-BCG-treated)	rs1801282	3p25.2	PPARG	G	C	0.07	Caucasian (White)	Leibovici et al. [33]
NMIBC Recurrence (TUR- and BCG-treated)	rs1799864 (corrected from author-reported rs391835*)*	3p21.31	CCR2	A	G	0.41	European (Southern Portugal)	Lima et al. [35]
NMIBC Recurrence (TUR- and BCG-treated)	rs5498	19p13.2	ICAM1	G	A	0.36	European (Southern Portugal)	Lima et al. [35]
NMIBC Recurrence (TUR- and BCG-treated)	rs2275913	6p12.2	IL17A	A	G	0.29	European (Southern Portugal)	Lima et al. [35]
NMIBC Recurrence (TUR- and BCG-treated)	rs13278062	8p21.3	TNFRSF10A	G	T	0.6	European (Southern Portugal)	Lima et al. [35]
NMIBC Recurrence (TUR- and BCG-treated)	rs3746162	19p13.3	GPX4	A	G	0.16	Caucasians (European decent)	Ke et al. [37]

NMIBC Recurrence (TUR- and BCG-treated)	rs7265992	20q11.22	GSS	A	G	0.19	Caucasians (European decent)	Ke et al. [37]
NMIBC Recurrence (TUR- and BCG-treated)	rs6060124	20q11.22	GSS	A	C	0.26	Caucasians (European decent)	Ke et al. [37]
NMIBC Recurrence (TUR- and BCG-treated)	rs7260770	20q11.22	GSS	A	G	0.22	Caucasians (European decent)	Ke et al. [37]
NMIBC Recurrence (TUR- and BCG-treated)	rs4911455	20q11.22	GSS	C	A	0.28	Caucasians (European decent)	Ke et al. [37]
NMIBC Recurrence (TUR-treated + Epirubicin)	rs2854509	19q13.31	XRCC1	A	C	0.82	Chinese	Deng et al. [38]
NMIBC Recurrence (TUR-treated + Epirubicin)	rs3213255	19q13.31	XRCC1	C	T	0.32	Chinese	Deng et al. [38]
NMIBC Recurrence (TUR-treated)	rs1042522	17p13.1	TP53	C	G	0.54	Japanese	Horikawa et al. [39]
NMIBC Recurrence (TUR-treated)	rs197412	1p13.2	DDX20	T	C	0.53	Caucasian (Northern American)	Ke et al. [40]
NMIBC Recurrence (TUR-treated)	rs12186785	5p13.3	DROSHA	C	T	0.05	Caucasian (Northern American)	Ke et al. [40]
NMIBC Recurrence-(BCG-treated)	rs804256	8p23.1	NEIL2	C	T	0.26	Caucasian (Northern American)	Wei et al. [16]
NMIBC Recurrence, NMIBC Recurrence (non-BCG-treated)	rs1052133	3p25.3	OGG1	G	C	0.3	Korean, Indian	Kim et al. [41], Gangwar et al. [14]
Recurrence	rs2042329	5q12.3	CWC27	T	G	0.34	Chinese	Wang et al. [42]
UBC Recurrence	rs2292016	5p13.1	OSMR	T	G	0.08	Chinese	Deng et al. [43]
UBC Recurrence	rs2278329	5p13.1	OSMR	A	G	0.08	Chinese	Deng et al. [43]
UBC Recurrence (BCG-treated)	rs4073	4q13.3	CXCL8	A	T	0.52	Indian	Ahirwar et al. [44]

BCG-Bacillus Calmette-Guérin; EA-effect allele; EAF-effect allele frequency; MIBC-muscle-invasive bladder cancer; NMIBC-non-muscle-invasive bladder cancer; RA-reference allele; SNP-single nucleotide polymorphism; TUR-transurethral resection; UBC-urinary bladder cancer.
*Global, based on 1000 Genomes Project.

References:

1. Ryk C, Koskela LR, Thiel T, Wiklund NP, Steineck G, Schumacher MC, de Verdier PJ. Outcome after BCG treatment for urinary bladder cancer may be influenced by polymorphisms in the NOS2 and NOS3 genes. Redox biology. 2015;6:272-7.
2. Wang M, Wang M, Yuan L, Wu D, Zhang Z, Yin C, Fu G, Wei Q, Zhang Z. A novel XPF -357A>C polymorphism predicts risk and recurrence of bladder cancer. Oncogene. 2010;29(13):1920-8.
3. Kiemeney LA, Sulem P, Besenbacher S, Vermeulen SH, Sigurdsson A, Thorleifsson G, Gudbjartsson DF, Stacey SN, Gudmundsson J, Zanon C, Kostic J, Masson G, Bjarnason H, Palsson ST, Skarphedinsson OB, Gudjonsson SA, Witjes JA, Grotenhuis AJ, Verhaegh GW, Bishop DT, Sak SC, Choudhury A, Elliott F, Barrett JH, Hurst CD, de Verdier PJ, Ryk C, Rudnai P, Gurzau E, Koppova K, Vineis P, Polidoro S, Guarrera S, Sacerdote C, Campagna M, Placidi D, Arici C, Zeegers MP, Kellen E, Gutierrez BS, Sanz-Velez JI, Sanchez-Zalabardo M, Valdivia G, Garcia-Prats MD, Hengstler JG, Blaszkewicz M, Dietrich H, Ophoff RA, van den Berg LH, Alexiusdottir K, Kristjansson K, Geirsson G, Nikulasson S, Petursdottir V, Kong A, Thorgeirsson T, Mungan NA, Lindblom A, van Es MA, Porru S, Buntinx F, Golka K, Mayordomo JI, Kumar R, Matullo G, Steineck G, Kiltie AE, Aben KKH, Jonsson E, Thorsteinsdottir U, Knowles MA, Rafnar T, Stefansson K. A sequence variant at 4p16.3 confers susceptibility to urinary bladder cancer. Nature genetics. 2010;42(5):415-9.
4. Zhou B, Zhang P, Tang T, Zhang K, Wang Y, Song Y, Liao H, Zhang L. Prognostic value of PDCD6 polymorphisms and the susceptibility to bladder cancer. Tumour biology : the journal of the International Society for Oncodevelopmental Biology and Medicine. 2014;35(8):7547-54.

5. Li Q, Tang T, Zhang P, Liu C, Pu Y, Zhang Y, Song H, Wang Y, Song Y, Su M, Zhou B, Zhang L. Correlation of IL-31 gene polymorphisms with susceptibility and clinical recurrence of bladder cancer. Familial cancer. 2018;17(4):577-85.
6. Zhang K, Zhou B, Zhang P, Zhang Z, Chen P, Pu Y, Song Y, Zhang L. Genetic variants in NAMPT predict bladder cancer risk and prognosis in individuals from southwest Chinese Han group. Tumour biology : the journal of the International Society for Oncodevelopmental Biology and Medicine. 2014;35(5):4031-40.
7. Wang M, Chu H, Li P, Yuan L, Fu G, Ma L, Shi D, Zhong D, Tong N, Qin C, Yin C, Zhang Z. Genetic variants in miRNAs predict bladder cancer risk and recurrence. Cancer research. 2012;72(23):6173-82.
8. Lee EK, Ye Y, Kamat AM, Wu X. Genetic variations in regulator of G-protein signaling (RGS) confer risk of bladder cancer. Cancer. 2013;119(9):1643-51.
9. Andrew AS, Gui J, Hu T, Wyszynski A, Marsit CJ, Kelsey KT, Schned AR, Tanyos SA, Pendleton EM, Ekstrom RM, Li Z, Zens MS, Borsuk M, Moore JH, Karagas MR. Genetic polymorphisms modify bladder cancer recurrence and survival in a USA population-based prognostic study. BJU international. 2015;115(2):238-47.
10. Yang X, Li P, Yang X, Qin C, Cao Q, Zhang Z, Wang M, Cai H, Gu J, Tao J, Gu M, Lu Q, Yin C. TSP-1-1223 A/G Polymorphism as a Potential Predictor of the Recurrence Risk of Bladder Cancer in a Chinese Population. International Journal of Genomics. 2013;2013:9.
11. Zhao H, Liang D, Grossman HB, Wu X. Glutathione peroxidase 1 gene polymorphism and risk of recurrence in patients with superficial bladder cancer. Urology. 2005;66(4):769-74.
12. Lin J, Dinney CP, Grossman HB, Jhamb M, Zhu Y, Spitz MR, Wu X. E-cadherin promoter polymorphism (C-160A) and risk of recurrence in patients with superficial bladder cancer. Clinical genetics. 2006;70(3):240-5.
13. Gangwar R, Mandhani A, Mittal RD. Caspase 9 and caspase 8 gene polymorphisms and susceptibility to bladder cancer in north Indian population. Annals of surgical oncology. 2009;16(7):2028-34.
14. Gangwar R, Ahirwar D, Mandhani A, Mittal RD. Do DNA repair genes OGG1, XRCC3 and XRCC7 have an impact on susceptibility to bladder cancer in the North Indian population? Mutation research. 2009;680(1-2):56-63.
15. Jaiswal PK, Goel A, Mandhani A, Mittal RD. Functional polymorphisms in promoter survivin gene and its association with susceptibility to bladder cancer in North Indian cohort. Molecular biology reports. 2012;39(5):5615-21.
16. Wei H, Kamat A, Chen M, Ke HL, Chang DW, Yin J, Grossman HB, Dinney CP, Wu X. Association of polymorphisms in oxidative stress genes with clinical outcomes for bladder cancer treated with Bacillus Calmette-Guerin. PloS one. 2012;7(6):e38533.
17. Decobert M, Larue H, Bergeron A, Harel F, Pfister C, Rousseau F, Lacombe L, Fradet Y. Polymorphisms of the human NRAMP1 gene are associated with response to bacillus Calmette-Guerin immunotherapy for superficial bladder cancer. The Journal of urology. 2006;175(4):1506-11.
18. Chiong E, Kesavan A, Mahendran R, Chan YH, Sng JH, Lim YK, Kamaraj R, Tan TM, Esuvaranathan K. NRAMP1 and hGPX1 gene polymorphism and response to bacillus Calmette-Guerin therapy for bladder cancer. Eur Urol. 2011;59(3):430-7.
19. Mittal RD, Singh R, Manchanda PK, Ahirwar D, Gangwar R, Kesarwani P, Mandhani A. XRCC1 codon 399 mutant allele: a risk factor for recurrence of urothelial bladder carcinoma in patients on BCG immunotherapy. Cancer biology & therapy. 2008;7(5):645-50.
20. Gu J, Zhao H, Dinney CP, Zhu Y, Leibovici D, Bermejo CE, Grossman HB, Wu X. Nucleotide excision repair gene polymorphisms and recurrence after treatment for superficial bladder cancer. Clinical cancer research : an official journal of the American Association for Cancer Research. 2005;11(4):1408-15.

21. Gangawar R, Ahirwar D, Mandhani A, Mittal RD. Impact of nucleotide excision repair ERCC2 and base excision repair APEX1 genes polymorphism and its association with recurrence after adjuvant BCG immunotherapy in bladder cancer patients of North India. Medical oncology (Northwood, London, England). 2010;27(2):159-66.
22. Ahirwar DK, Agrahari A, Mandhani A, Mittal RD. Cytokine gene polymorphisms are associated with risk of urinary bladder cancer and recurrence after BCG immunotherapy. Biomarkers : biochemical indicators of exposure, response, and susceptibility to chemicals. 2009;14(4):213-8.
23. Gangwar R, Mandhani A, Mittal RD. XPC gene variants: a risk factor for recurrence of urothelial bladder carcinoma in patients on BCG immunotherapy. Journal of cancer research and clinical oncology. 2010;136(5):779-86.
24. Chen M, Hildebrandt MA, Clague J, Kamat AM, Picornell A, Chang J, Zhang X, Izzo J, Yang H, Lin J, Gu J, Chanock S, Kogevinas M, Rothman N, Silverman DT, Garcia-Closas M, Grossman HB, Dinney CP, Malats N, Wu X. Genetic variations in the sonic hedgehog pathway affect clinical outcomes in non-muscle-invasive bladder cancer. Cancer prevention research (Philadelphia, Pa). 2010;3(10):1235-45.
25. Srivastava P, Kapoor R, Mittal RD. Association of single nucleotide polymorphisms in promoter of matrix metalloproteinase-2, 8 genes with bladder cancer risk in Northern India. Urologic oncology. 2013;31(2):247-54.
26. Jaiswal PK, Singh V, Srivastava P, Mittal RD. Association of IL-12, IL-18 variants and serum IL-18 with bladder cancer susceptibility in North Indian population. Gene. 2013;519(1):128-34.
27. Deng X, Yang X, Cheng Y, Liu X, Li X, Zhao R, Qin C, Lu Q, Yin C. GSTP1 and GSTO1 single nucleotide polymorphisms and the response of bladder cancer patients to intravesical chemotherapy. Scientific reports. 2015;5:14000.
28. Williams SB, Kamat AM, Mmeje C, Ye Y, Huang M, Chang DW, Dinney CP, Wu X. Genetic variants in the inflammation pathway as predictors of recurrence and progression in non-muscle invasive bladder cancer treated with Bacillus Calmette-Guerin. Oncotarget. 2017;8(51):88782-91.
29. Wang Z, Lim YK, Lim HCC, Chan YH, Ngiam N, Raman Nee Mani L, Esuvaranathan K, Ng CF, Teoh J, Chan E, Mahendran R, Chiong E. The Role of Vitamin D Receptor Polymorphisms in Predicting the Response to Therapy for Nonmuscle Invasive Bladder Carcinoma. The Journal of urology. 2018;200(4):737-42.
30. Gangwar R, Mittal RD. Association of selected variants in genes involved in cell cycle and apoptosis with bladder cancer risk in North Indian population. DNA and cell biology. 2010;29(7):349-56.
31. Xie L, Sun Y, Chen T, Tian D, Li Y, Zhang Y, Ding N, Shen Z, Xu H, Nian X, Sha N, Han R, Hu H, Wu C. Association between MDM2 SNP309 T>G polymorphism and the risk of bladder cancer: new data in a Chinese population and an updated meta-analysis. OncoTargets and therapy. 2015;8:3679-90.
32. Ahirwar D, Kesarwani P, Manchanda PK, Mandhani A, Mittal RD. Anti- and proinflammatory cytokine gene polymorphism and genetic predisposition: association with smoking, tumor stage and grade, and bacillus Calmette-Guerin immunotherapy in bladder cancer. Cancer genetics and cytogenetics. 2008;184(1):1-8.
33. Leibovici D, Grossman HB, Dinney CP, Millikan RE, Lerner S, Wang Y, Gu J, Dong Q, Wu X. Polymorphisms in inflammation genes and bladder cancer: from initiation to recurrence, progression, and survival. Journal of clinical oncology : official journal of the American Society of Clinical Oncology. 2005;23(24):5746-56.
34. Ahirwar DK, Mandhani A, Dharaskar A, Kesarwani P, Mittal RD. Association of tumour necrosis factor-alpha gene (T-1031C, C-863A, and C-857T) polymorphisms with bladder cancer susceptibility and outcome after bacille Calmette-Guerin immunotherapy. BJU international. 2009;104(6):867-73.

35. Lima L, Oliveira D, Ferreira JA, Tavares A, Cruz R, Medeiros R, Santos L. The role of functional polymorphisms in immune response genes as biomarkers of bacille Calmette-Guerin (BCG) immunotherapy outcome in bladder cancer: establishment of a predictive profile in a Southern Europe population. BJU international. 2015;116(5):753-63.
36. Li P, Zhang X, Deng X, Tao J, Qin C, Yang X, Cheng Y, Lu Q, Wang Z, Yin C. Pharmacogenetic association between XRCC1 polymorphisms and improved outcomes in bladder cancer patients following intravesical instillation of epirubicin. International journal of clinical and experimental medicine. 2015;8(7):11167-73.
37. Ke HL, Lin J, Ye Y, Wu WJ, Lin HH, Wei H, Huang M, Chang DW, Dinney CP, Wu X. Genetic Variations in Glutathione Pathway Genes Predict Cancer Recurrence in Patients Treated with Transurethral Resection and Bacillus Calmette-Guerin Instillation for Non-muscle Invasive Bladder Cancer. Annals of surgical oncology. 2015;22(12):4104-10.
38. Deng X, Zhang X, Cheng Y, Yang X, Zhao R, Liu X, Li X, Qin C, Lu Q, Yin C. XRCC1 polymorphisms associated with survival among Chinese bladder cancer patients receiving epirubicin and mitomycin C. Tumour biology : the journal of the International Society for Oncodevelopmental Biology and Medicine. 2015;36(6):4591-6.
39. Horikawa Y, Nadaoka J, Saito M, Kumazawa T, Inoue T, Yuasa T, Tsuchiya N, Nishiyama H, Ogawa O, Habuchi T. Clinical implications of the MDM2 SNP309 and p53 Arg72Pro polymorphisms in transitional cell carcinoma of the bladder. Oncology reports. 2008;20(1):49-55.
40. Ke HL, Chen M, Ye Y, Hildebrandt MA, Wu WJ, Wei H, Huang M, Chang DW, Dinney CP, Wu X. Genetic variations in micro-RNA biogenesis genes and clinical outcomes in non-muscle-invasive bladder cancer. Carcinogenesis. 2013;34(5):1006-11.
41. Kim EJ, Jeong P, Quan C, Kim J, Bae SC, Yoon SJ, Kang JW, Lee SC, Jun Wee J, Kim WJ. Genotypes of TNF-alpha, VEGF, hOGG1, GSTM1, and GSTT1: useful determinants for clinical outcome of bladder cancer. Urology. 2005;65(1):70-5.
42. Wang M, Li Z, Chu H, Lv Q, Ye D, Ding Q, Xu C, Guo J, Du M, Chen J, Song Z, Yin C, Qin C, Gu C, Zhu Y, Xia G, Liu F, Zhang Z, Yuan L, Fu G, Hu Z, Tong N, Shen J, Liu K, Sun J, Sun Y, Li J, Li X, Shen H, Xu J, Shi Y, Zhang Z. Genome-Wide Association Study of Bladder Cancer in a Chinese Cohort Reveals a New Susceptibility Locus at 5q12.3. Cancer research. 2016;76(11):3277-84.
43. Deng S, He SY, Zhao P, Zhang P. The role of oncostatin M receptor gene polymorphisms in bladder cancer. World journal of surgical oncology. 2019;17(1):30.
44. Ahirwar DK, Mandhani A, Mittal RD. IL-8 -251 T > A polymorphism is associated with bladder cancer susceptibility and outcome after BCG immunotherapy in a northern Indian cohort. Archives of medical research. 2010;41(2):97-103.
