# Supplementary material for: External Replication of Urinary Bladder Cancer Prognostic Polymorphisms in the UK Biobank
Source: Front Oncol. 2019 Oct 18;9:1082. doi: 10.3389/fonc.2019.01082 (PMC6813571; doi:10.3389/fonc.2019.01082)
Supplement: Supplementary file 2 [file Data_Sheet_2.PDF]

Supplementary Table 2. Previously reported polymorphisms in association with bladder cancer death.

Outcome	SNP	Locus	Gene	EA	RA	EAF*	Discovery population	References
High-risk NMIBC Cancer-specific death (BCG-treated)	rs1799983	7q36.1	NOS3	G	T	0.82	European (Sweden)	Ryk et al. [1]
MIBC Cancer-specific death	rs1800795	7p15.3	IL6	C	G	0.14	Caucasian (White)	Leibovici et al.[2]
MIBC Cancer-specific death	rs334358	9q22.33	TGFBR1	T	G	0.12	European (Spain)	Castillejo et al. [3]
MIBC Cancer-specific death	rs868	9q22.33	TGFBR1	G	A	0.12	European (Spain)	Castillejo et al. [3]
MIBC Cancer-specific death (Cystectomy-treated)	rs1042522	17p13.1	TP53	C	G	0.54	Japanese	Horikawa et al. [4]
MIBC Cancer-specific death (Radiotherapy-treated)	rs7180135	15q15.1	RAD51	G	A	0.27	European (United Kingdom)	Teo et al. [5]
MIBC Cancer-specific death (Radiotherapy-treated)	rs1805363	11q21	MRE11	A	G	0.03	European (Denmark)	Teo et al. [6]
MIBC Overall survival	rs9906827	17q25.3	RPTOR	A	G	0.42	Caucasian (Northern American)	Chen et al. [7]
MIBC Overall survival	rs1051013	9q32	RGS3	A/C	T	0.84	Caucasian (Northern American)	Lee et al. [8]
MIBC Overall survival	rs1395960	1q23.3	RGS5	A	G	0.24	Caucasian (Northern American)	Lee et al. [8]
MIBC Overall survival	rs762861	4p16.3	RGS12	C	G	0.36	Caucasian (Northern American)	Lee et al. [8]
MIBC Overall survival	rs17855750	16p12.1-p11.2	IL27	G	T	0.07	Chinese	Zhou et al. [9]
MIBC Overall survival	rs10515074	5q13.1	PIK3R1	G	A	0.2	Caucasian (Northern American)	Chen et al. [7]
MIBC Overall survival	rs3730050	19q13.2	AKT2	A	G	0.25	Caucasian (Northern American)	Chen et al. [7]
MIBC Overall survival	rs2344673	1q23.3	RGS5	A	G	0.05	Caucasian (Northern American)	Lee et al. [8]
MIBC Overall survival	rs10917690	1q23.3	RGS5	G	A	0.29	Caucasian (Northern American)	Lee et al. [8]
MIBC Overall survival	rs1890398	1q31.2	RGS2	C	T	0.56	Caucasian (Northern American)	Lee et al. [8]
MIBC Overall survival	rs12035879	1q23.3	RGS5	G	A	0.68	Caucasian (Northern American)	Lee et al. [8]
MIBC Overall survival	rs10753605	1q23.3	RGS5	C	T	0.35	Caucasian (Northern American)	Lee et al. [8]
MIBC Overall survival	rs156697	10q25.1	GSTO2	G	A	0.56	European (Serbia)	Djukic et al. [10]
MIBC Overall survival	rs4925	10q25.1	GSTO1	A	C	0.18	European (Serbia)	Djukic et al. [10]
MIBC Overall survival (Platinum-based chemotherapy-treated)	rs11615	19q13.32	ERCC1	T	C	0.33	Chinese	Xu et al. [11]
NMIBC Overall survival	rs2662238	5q14.2	XRCC4	A	G	0.36	Caucasian (Northern American)	Andrew et al. [12]

NMIBC Overall survival	rs4987059	11p15.5	DRD4	A	G	0.04	Caucasian (Northern American)	Andrew et al. [12]
NMIBC Overall survival	rs3756712	5p15.33	PDCD6	G	T	0.4	Chinese	Zhou et al. [13]
NMIBC Overall survival	rs2292016	5p13.1	OSMR	T	G	0.08	Chinese	Deng et al. [14]
UBC Cancer-specific death	rs2279744	12q15	MDM2	G	T	0.37	Japanese	Shinohara et al. [15]
UBC Cancer-specific death	rs4129009	4p14	TLR10	T	C	0.85	European (Spain)	Guirado et al. [16]
UBC Cancer-specific death	rs1801133	1p36.22	MTHFR	T	C	0.25	European (Sweden)	Sanyal et al. [17]
UBC Cancer-specific death	rs5443	12p13.31	GNB3	T	C	0.49	European (Germany)	Eisenhardt et al. [18]
UBC Cancer-specific death	rs9302752	16q12.1	NOD2	A	G	0.5	European (Spain)	Guirado et al. [16]
UBC Overall survival	rs13181	19q13.3	ERCC2	C	A	0.24	European (Sweden)	Sanyal et al. [19]
UBC Overall survival	rs2854461	1q42.12	EPHX1	C	A	0.64	Caucasian (Northern American)	Andrew et al. [20]
UBC Overall survival	rs2279115	18q21.33	BCL2	A	C	0.39	European (Germany)	Hess et al. [21]
UBC Overall survival	rs11543848 (merged into rs2227983)	7p11.2	EGFR	A	G	0.29	Caucasian (Northern American)	Mason et al. [22]
UBC Overall survival	rs2017000	7p11.2	EGFR	G	A	0.33	Caucasian (Northern American)	Mason et al. [22]
UBC Overall survival	rs4986998 (merged into rs1131341)	16q22.1	NQO1	T	C	0.02	European (Sweden)	Sanyal et al. [17]
UBC Overall survival	rs6024840	20q13.2	AURKA	C	T	0.45	Caucasian (Northern American)	Andrew et al. [20]
UBC Overall survival	rs1042640	2q37.1	UGT1A1	C	G	0.82	Caucasian (Northern American)	Andrew et al. [20]
UBC Overall survival	rs1126579	2q35	CXCR2	C	T	0.6	Caucasian (Northern American)	Andrew et al. [20]
UBC Overall survival	rs528778	10p14	GATA3	T	C	0.14	Caucasian (Northern American)	Andrew et al. [20]
UBC Overall survival	rs1800067	16p13.12	ERCC4	A	G	0.03	Caucasian (Northern American)	Andrew et al. [20]
UBC Overall survival	rs1994251	20q11.21	BCL2L1	C	A	0.24	Caucasian (Northern American)	Andrew et al. [20]
UBC Overall survival	rs9282638	3q13.33	CD80	A	G	0.87	Caucasian (Northern American)	Andrew et al. [20]
UBC Overall survival	rs35402311	8q11.23	RB1CC1	T	C	0.02	Caucasian (Northern American)	Andrew et al. [20]
UBC Overall survival	rs1801018	18q21.33	BCL2	A	G	0.76	European (Germany)	Hess et al. [21]
UBC Overall survival	rs2293347	7p11.2	EGFR	T	C	0.14	Caucasian (Northern American)	Mason et al. [22]

UBC Overall survival (Chemotherapy-treated)	rs915927	19q13.2	XRCC1	G	A	0.32	European (Italy)	Sacerdote et al. [23]
UBC Overall survival (Chemotherapy-treated)	rs762507	19q13.2	XRCC1	A	G	0.29	European (Italy)	Sacerdote et al. [23]
UBC Overall survival (Chemotherapy-treated)	rs2854501	19q13.2	XRCC1	T	C	0.82	European (Italy)	Sacerdote et al. [23]
UBC Overall survival (Chemotherapy-treated)	rs2854509	19q13.2	XRCC1	A	C	0.18	European (Italy)	Sacerdote et al. [23]
UBC Overall survival (Chemotherapy-treated)	rs3213255	19q13.2	XRCC1	C	T	0.32	European (Italy)	Sacerdote et al. [23]
UBC Overall survival (Chemotherapy-treated)	rs171140	19q13.3	ERCC2	C	A	0.37	European (Italy)	Sacerdote et al. [23]

BCG-Bacillus Calmette-Guérin; EA-effect allele; EAF-effect allele frequency; MIBC-muscle-invasive bladder cancer; NMIBC-non-muscle-invasive bladder cancer; RA-reference allele; SNP-single nucleotide polymorphism; TUR-transurethral resection; UBC-urinary bladder cancer.

*Global, based on 1000 Genomes Project.

References:

1. Ryk C, Koskela LR, Thiel T, Wiklund NP, Steineck G, Schumacher MC, de Verdier PJ. Outcome after BCG treatment for urinary bladder cancer may be influenced by polymorphisms in the NOS2 and NOS3 genes. Redox biology. 2015;6:272-7.
2. Leibovici D, Grossman HB, Dinney CP, Millikan RE, Lerner S, Wang Y, Gu J, Dong Q, Wu X. Polymorphisms in inflammation genes and bladder cancer: from initiation to recurrence, progression, and survival. Journal of clinical oncology : official journal of the American Society of Clinical Oncology. 2005;23(24):5746-56.
3. Castillejo A, Rothman N, Murta-Nascimento C, Malats N, Garcia-Closas M, Gomez-Martinez A, Lloreta J, Tardon A, Serra C, Garcia-Closas R, Chanock S, Silverman DT, Dosemeci M, Kogevinas M, Carrato A, Soto JL, Real FX. TGFB1 and TGFBR1 polymorphic variants in relationship to bladder cancer risk and prognosis. International journal of cancer. 2009;124(3):608-13.
4. Horikawa Y, Nadaoka J, Saito M, Kumazawa T, Inoue T, Yuasa T, Tsuchiya N, Nishiyama H, Ogawa O, Habuchi T. Clinical implications of the MDM2 SNP309 and p53 Arg72Pro polymorphisms in transitional cell carcinoma of the bladder. Oncology reports. 2008;20(1):49-55.
5. Teo MT, Landi D, Taylor CF, Elliott F, Vaslin L, Cox DG, Hall J, Landi S, Bishop DT, Kiltie AE. The role of microRNA-binding site polymorphisms in DNA repair genes as risk factors for bladder cancer and breast cancer and their impact on radiotherapy outcomes. Carcinogenesis. 2012;33(3):581-6.
6. Teo MT, Dyrskjot L, Nsengimana J, Buchwald C, Snowden H, Morgan J, Jensen JB, Knowles MA, Taylor G, Barrett JH, Borre M, Orntoft TF, Bishop DT, Kiltie AE. Next-generation sequencing identifies germline MRE11A variants as markers of radiotherapy outcomes in muscle-invasive bladder cancer. Annals of oncology : official journal of the European Society for Medical Oncology. 2014;25(4):877-83.
7. Chen M, Gu J, Delclos GL, Killary AM, Fan Z, Hildebrandt MA, Chamberlain RM, Grossman HB, Dinney CP, Wu X. Genetic variations of the PI3K-AKT-mTOR pathway and clinical outcome in muscle invasive and metastatic bladder cancer patients. Carcinogenesis. 2010;31(8):1387-91.

8. Lee EK, Ye Y, Kamat AM, Wu X. Genetic variations in regulator of G-protein signaling (RGS) confer risk of bladder cancer. Cancer. 2013;119(9):1643-51.
9. Zhou B, Zhang P, Tang T, Liao H, Zhang K, Pu Y, Chen P, Song Y, Zhang L. Polymorphisms and plasma levels of IL-27: impact on genetic susceptibility and clinical outcome of bladder cancer. BMC cancer. 2015;15:433.
10. Djukic TI, Savic-Radojevic AR, Pekmezovic TD, Matic MG, Pljesa-Ercegovac MS, Coric VM, Radic TM, Suvakov SR, Krivic BN, Dragicevic DP, Simic TP. Glutathione S-transferase T1, O1 and O2 polymorphisms are associated with survival in muscle invasive bladder cancer patients. PloS one. 2013;8(9):e74724.
11. Xu ZC, Cai HZ, Li X, Xu WZ, Xu T, Yu B, Zou Q, Xu L. ERCC1 C118T polymorphism has predictive value for platinum-based chemotherapy in patients with late-stage bladder cancer. Genetics and molecular research : GMR. 2016;15(2).
12. Andrew AS, Gui J, Hu T, Wyszynski A, Marsit CJ, Kelsey KT, Schned AR, Tanyos SA, Pendleton EM, Ekstrom RM, Li Z, Zens MS, Borsuk M, Moore JH, Karagas MR. Genetic polymorphisms modify bladder cancer recurrence and survival in a USA population-based prognostic study. BJU international. 2015;115(2):238-47.
13. Zhou B, Zhang P, Tang T, Zhang K, Wang Y, Song Y, Liao H, Zhang L. Prognostic value of PDCD6 polymorphisms and the susceptibility to bladder cancer. Tumour biology : the journal of the International Society for Oncodevelopmental Biology and Medicine. 2014;35(8):7547-54.
14. Deng S, He SY, Zhao P, Zhang P. The role of oncostatin M receptor gene polymorphisms in bladder cancer. World journal of surgical oncology. 2019;17(1):30.
15. Shinohara A, Sakano S, Hinoda Y, Nishijima J, Kawai Y, Misumi T, Nagao K, Hara T, Matsuyama H. Association of TP53 and MDM2 polymorphisms with survival in bladder cancer patients treated with chemoradiotherapy. Cancer science. 2009;100(12):2376-82.
16. Guirado M, Gil H, Saenz-Lopez P, Reinboth J, Garrido F, Cozar JM, Ruiz-Cabello F, Carretero R. Association between C13ORF31, NOD2, RIPK2 and TLR10 polymorphisms and urothelial bladder cancer. Human immunology. 2012;73(6):668-72.
17. Sanyal S, Ryk C, De Verdier PJ, Steineck G, Larsson P, Onelov E, Hemminki K, Kumar R. Polymorphisms in NQO1 and the clinical course of urinary bladder neoplasms. Scandinavian journal of urology and nephrology. 2007;41(3):182-90.
18. Eisenhardt A, Siffert W, Rosskopf D, Musch M, Mosters M, Roggenbuck U, Jockel KH, Rubben H, Lummen G. Association study of the G-protein beta3 subunit C825T polymorphism with disease progression in patients with bladder cancer. World journal of urology. 2005;23(4):279-86.
19. Sanyal S, De Verdier PJ, Steineck G, Larsson P, Onelov E, Hemminki K, Kumar R. Polymorphisms in XPD, XPC and the risk of death in patients with urinary bladder neoplasms. Acta oncologica (Stockholm, Sweden). 2007;46(1):31-41.
20. Andrew AS, Gui J, Sanderson AC, Mason RA, Morlock EV, Schned AR, Kelsey KT, Marsit CJ, Moore JH, Karagas MR. Bladder cancer SNP panel predicts susceptibility and survival. Human genetics. 2009;125(5-6):527-39.
21. Hess J, Stelmach P, Eisenhardt A, Rubben H, Reis H, Schmid KW, Bachmann HS. Impact of BCL2 polymorphisms on survival in transitional cell carcinoma of the bladder. Journal of cancer research and clinical oncology. 2017;143(9):1659-70.
22. Mason RA, Morlock EV, Karagas MR, Kelsey KT, Marsit CJ, Schned AR, Andrew AS. EGFR pathway polymorphisms and bladder cancer susceptibility and prognosis. Carcinogenesis. 2009;30(7):1155-60.
23. Sacerdote C, Guarrera S, Ricceri F, Pardini B, Polidoro S, Allione A, Critelli R, Russo A, Andrew AS, Ye Y, Wu X, Kiemeney LA, Bosio A, Casetta G, Cucchiarale G, Destefanis P, Gontero P, Rolle L, Zitella A, Fontana D, Vineis P, Matullo G. Polymorphisms in the XRCC1 gene modify survival of bladder cancer patients treated with chemotherapy. International journal of cancer. 2013;133(8):2004-9.
