# Supplementary material for: External Replication of Urinary Bladder Cancer Prognostic Polymorphisms in the UK Biobank
Source: Front Oncol. 2019 Oct 18;9:1082. doi: 10.3389/fonc.2019.01082 (PMC6813571; doi:10.3389/fonc.2019.01082)
Supplement: Supplementary file 3 [file Data_Sheet_3.PDF]

Supplementary Table 3. Previously reported polymorphisms in association with age at the time of bladder cancer diagnosis.

Outcome	SNP	Locus	Gene	EA	RA	EAF*	Discovery population	References
Age, years	rs798766	4p16.3	TACC3/FGFR3	T	C	0.24	European (Multiple)	Kiemeney et al. [1]
Age (≥50 years)	rs25487	19q13.2	XRCC1	A	G	0.26	Caucasian (Northern American)	Kelsey et al. [2]
Age (>56 years)	rs710521	3q28	TP63	A	G	0.8	Caucasian (Northern American)	Stern et al. [3]
Age (>60 years)	rs874945	12q13.13	HOTAIR	A	G	0.36	Chinese	Wang et al. [4]
Age (>60 years)	rs710886	8q24.21	PCAT1	G	A	0.47	Chinese	Lin et al. [5]
Age (≤60 years)	rs217727	11p15.5	H19 (lncRNA)	A	G	0.2	Chinese	Hua et al. [6]
Age (>65 years)	rs1052133	3p25.3	OGG1	G	C	0.3	Chinese	Ma et al. [7]
Age (>65 years)	rs884225	7p11.2	EGFR	C	T	0.19	Chinese	Chu et al. [8]
Age (>65 years)	rs7003908	8q11.21	PRKDC	T	G	0.33	Chinese	Wang et al. [9]
Age (>65 years)	rs1057868	7q11.23	POR	T	C	0.29	Chinese	Xiao et al. [10]
Age (≤65 years / Healthy controls)	rs9642880	8q24.21	CASC11	T	G	0.54	Chinese	Wang et al. [11]
Age (≥65 years / Healthy controls)	rs9344	11q13.3	CCND1	A	G	0.41	Chinese	Yuan et al. [12]
Age (</≥70.2)	rs41515546	7q31.33	-	C	T	0.14	European	Lipunova et al. [13]
Age (</≥70.2)	rs17149636	7q31.33	-	G	A	0.14	European	Lipunova et al. [13]
Age (</≥70.2)	rs17149628	7q31.33	-	T	C	0.14	European	Lipunova et al. [13]
Age (</≥70.2)	rs12666814	7q31.33	-	T	C	0.14	European	Lipunova et al. [13]
Age (</≥70.2)	rs73223045	7q31.33	-	C	G	0.14	European	Lipunova et al. [13]
Age (</≥70.2)	rs12673089	7q31.33	-	T	C	0.14	European	Lipunova et al. [13]
Age (</≥70.2)	rs17149580	7q31.33	-	G	A	0.14	European	Lipunova et al. [13]
Age (</≥70.2)	rs17149630	7q31.33	-	T	C	0.14	European	Lipunova et al. [13]

EA-effect allele; EAF-effect allele frequency; MIBC-muscle-invasive bladder cancer; NMIBC-non-muscle-invasive bladder cancer; RA-reference allele; SNP-single nucleotide polymorphism

*Global, based on 1000 Genomes Project.

1. Kiemeney LA, Sulem P, Besenbacher S, Vermeulen SH, Sigurdsson A, Thorleifsson G, Gudbjartsson DF, Stacey SN, Gudmundsson J, Zanon C, Kostic J, Masson G, Bjarnason H, Palsson ST, Skarphedinsson OB, Gudjonsson SA, Witjes JA, Grotenhuis AJ, Verhaegh GW, Bishop DT, Sak SC, Choudhury A, Elliott F, Barrett JH, Hurst CD, de Verdier PJ, Ryk C, Rudnai P, Gurzau E, Koppova K, Vineis P, Polidoro S, Guarrera S, Sacerdote C, Campagna M, Placidi D, Arici C, Zeegers MP, Kellen E, Gutierrez BS, Sanz-Velez JI, Sanchez-Zalabardo M, Valdivia G, Garcia-Prats MD, Hengstler JG, Blaszkewicz M, Dietrich H, Ophoff RA, van den Berg LH, Alexiusdottir K, Kristjansson K, Geirsson G, Nikulasson S, Petursdottir V, Kong A, Thorgeirsson T, Mungan NA, Lindblom A, van Es MA, Porru S, Buntinx F, Golka K, Mayordomo JI, Kumar R, Matullo G, Steineck G, Kiltie AE, Aben KKH, Jonsson E, Thorsteinsdottir U, Knowles MA, Rafnar T, Stefansson K. A sequence variant at 4p16.3 confers susceptibility to urinary bladder cancer. Nature genetics. 2010;42(5):415-9.
2. Kelsey KT, Park S, Nelson HH, Karagas MR. A population-based case-control study of the XRCC1 Arg399Gln polymorphism and susceptibility to bladder cancer. Cancer epidemiology, biomarkers & prevention : a publication of the American Association for Cancer Research, cosponsored by the American Society of Preventive Oncology. 2004;13(8):1337-41.
3. Stern MC, Van Den Berg D, Yuan JM, Conti DV, Gago-Dominguez M, Pike MC, Xiang YB, Gao YT, Cortessis VK. Sequence variant on 3q28 and urinary bladder cancer risk: findings from the Los Angeles-Shanghai bladder case-control study. Cancer epidemiology, biomarkers & prevention : a publication of the American Association for Cancer Research, cosponsored by the American Society of Preventive Oncology. 2009;18(11):3057-61.
4. Wang X, Wang W, Zhang Q, Gu D, Zhang K, Ge Y, Chu H, Du M, Xu B, Wang M, Lv X, Zhang Z, Yuan L, Gong W. Tagging SNPs in the HOTAIR gene are associated with bladder cancer risk in a Chinese population. Gene. 2018;664:22-6.
5. Lin Y, Ge Y, Wang Y, Ma G, Wang X, Liu H, Wang M, Zhang Z, Chu H. The association of rs710886 in lncRNA PCAT1 with bladder cancer risk in a Chinese population. Gene. 2017;627:226-32.
6. Hua Q, Lv X, Gu X, Chen Y, Chu H, Du M, Gong W, Wang M, Zhang Z. Genetic variants in lncRNA H19 are associated with the risk of bladder cancer in a Chinese population. Mutagenesis. 2016;31(5):531-8.
7. Ma L, Chu H, Wang M, Shi D, Zhong D, Li P, Tong N, Yin C, Zhang Z. hOGG1 Ser326Cys polymorphism is associated with risk of bladder cancer in a Chinese population: a case-control study. Cancer science. 2012;103(7):1215-20.
8. Chu H, Wang M, Jin H, Lv Q, Wu D, Tong N, Ma L, Shi D, Zhong D, Fu G, Yuan L, Qin C, Yin C, Zhang Z. EGFR 3'UTR 774T>C polymorphism contributes to bladder cancer risk. Mutagenesis. 2013;28(1):49-55.
9. Wang SY, Peng L, Li CP, Li AP, Zhou JW, Zhang ZD, Liu QZ. Genetic variants of the XRCC7 gene involved in DNA repair and risk of human bladder cancer. International journal of urology : official journal of the Japanese Urological Association. 2008;15(6):534-9.
10. Xiao X, Ma G, Li S, Wang M, Liu N, Ma L, Zhang Z, Chu H, Zhang Z, Wang SL. Functional POR A503V is associated with the risk of bladder cancer in a Chinese population. Scientific reports. 2015;5:11751.
11. Wang M, Wang M, Zhang W, Yuan L, Fu G, Wei Q, Zhang Z. Common genetic variants on 8q24 contribute to susceptibility to bladder cancer in a Chinese population. Carcinogenesis. 2009;30(6):991-6.
12. Yuan L, Gu X, Shao J, Wang M, Wang M, Zhu Q, Zhang Z. Cyclin D1 G870A polymorphism is associated with risk and clinicopathologic characteristics of bladder cancer. DNA and cell biology. 2010;29(10):611-7.
13. Lipunova N, Wesselius A, Cheng KK, van Schooten F-J, Bryan RT, Cazier J-B, Galesloot TE, Kiemeney LALM, Zeegers MP. Genome-wide Association Study for Tumour Stage, Grade, Size, and Age at Diagnosis of Non–muscle-invasive Bladder Cancer. European Urology Oncology.
