# Supplementary material for: External Replication of Urinary Bladder Cancer Prognostic Polymorphisms in the UK Biobank
Source: Front Oncol. 2019 Oct 18;9:1082. doi: 10.3389/fonc.2019.01082 (PMC6813571; doi:10.3389/fonc.2019.01082)
Supplement: Supplementary file 4 [file Data_Sheet_4.PDF]

Supplementary Table 4. Previously reported polymorphisms in association with bladder cancer progression.

Outcome	SNP	Locus	Gene	EA	RA	EAF*	Discovery population	References
NMIBC Progression	rs17350793 (has since merged into rs12628)	11p15.5	HRAS	C	T	0.30	European (Sweden)	Sanyal et al. [1]
UBC Progression	rs2297518	17q11.2	NOS2	T	C	0.17	European (Sweden)	Ryk et al. [2]
NMIBC Progression	rs10917690	1q23.3	RGS5	G	A	0.29	Caucasian (Northern American)	Lee et al. [3]
NMIBC Progression	rs4075958	5q35.3	RGS14	A	G	0.18	Caucasian (Northern American)	Lee et al. [3]
NMIBC Progression	rs10926466	1q23.1	RGS7	T	C	0.63	Caucasian (Northern American)	Lee et al. [3]
NMIBC Progression	rs12038803	1q23.1	RGS7	C/G/T? (Not reported)	A	0.66	Caucasian (Northern American)	Lee et al. [3]
High-risk NMIBC Progression (BCG-treated)	rs2070744	7q36.1	NOS3	T	C	0.77	European (Sweden)	Ryk et al. [4]
High-risk NMIBC Progression (BCG-treated)	rs1799983	7q36.1	NOS3	G	T	0.82	European (Sweden)	Ryk et al. [4]
UBC Progression (metastases present)	rs2910164	5q33.3	MIR146A	G	C	0.32 (TOPMED)	Chinese	Deng et al. [5]
NMIBC Progression	rs3088440	9p21	CDKN2A	T	C	0.17	European (Sweden)	Sakano et al. [6]
NMIBC progression (BCG-treated)	rs1800896	1q32.1	IL10	G	A	0.27	Turkish	Basturk et al. [7]
NMIBC progression (BCG-treated)	rs2243248	5q31.1	IL4	G	T	0.11	Turkish	Basturk et al. [7]
NMIBC progression (BCG-treated)	rs1800470	19q13.2	TGFB1	T	C	0.55	Turkish	Basturk et al. [7]
NMIBC progression (BCG-treated)	rs1800471	19q13.2	TGFB1	G	C	0.05	Turkish	Basturk et al. [7]
Progression (metastases present)	rs9302752	16q12.1	NOD2	G	A	0.50	European (Spain)	Guirado et al. [8]
NMIBC Progression	rs3890995	12q24.11	UNG	C	T	0.22	Caucasian (Northern American)	Wei et al. [9]
NMIBC Progression (TUR-treated)	rs720012	22q11.21	DGCR8	A	G	0.22	Caucasian (Northern American)	Ke et al. [10]
NMIBC Progression (TUR-treated)	rs2073778	22q11.21	DGCR8	T	C	0.22	Caucasian (Northern American)	Ke et al. [10]
NMIBC Progression	rs1323291	1q31.2	RGS1	C	A	0.15	Caucasian (Northern American)	Lee et al. [3]
NMIBC Progression	rs6678136	1q23.3	RGS4	A	G	0.46	Caucasian (Northern American)	Lee et al. [3]
NMIBC Progression	rs11585883	1q23.3	RGS5	C	T	0.03	Caucasian (Northern American)	Lee et al. [3]
MIBC Progression	rs11615	19q13.32	ERCC1	T	C	0.33	Chinese	Xu et al. [11]

UBC Progression	rs1801018	18q21.33	BCL2	A	G	0.76	European (Germany)	Hess et al. [12]

BCG-Bacillus Calmette-Guérin; EA-effect allele; EAF-effect allele frequency; MIBC-muscle-invasive bladder cancer; NMIBC-non-muscle-invasive bladder cancer; RA-reference allele; SNP-single nucleotide polymorphism; TUR-transurethral resection; UBC-urinary bladder cancer.
*Global, based on 1000 Genomes Project (unless otherwise specified).

References:

1. Sanyal S, De Verdier PJ, Steineck G, Larsson P, Onelov E, Hemminki K, Kumar R. Polymorphisms in XPD, XPC and the risk of death in patients with urinary bladder neoplasms. Acta oncologica (Stockholm, Sweden). 2007;46(1):31-41.
2. Ryk C, Wiklund NP, Nyberg T, De Verdier PJ. Ser608Leu polymorphisms in the nitric oxide synthase-2 gene may influence urinary bladder cancer pathogenesis. Scandinavian journal of urology and nephrology. 2011;45(5):319-25.
3. Lee EK, Ye Y, Kamat AM, Wu X. Genetic variations in regulator of G-protein signaling (RGS) confer risk of bladder cancer. Cancer. 2013;119(9):1643-51.
4. Ryk C, Koskela LR, Thiel T, Wiklund NP, Steineck G, Schumacher MC, de Verdier PJ. Outcome after BCG treatment for urinary bladder cancer may be influenced by polymorphisms in the NOS2 and NOS3 genes. Redox biology. 2015;6:272-7.
5. Deng S, Wang W, Li X, Zhang P. Common genetic polymorphisms in pre-microRNAs and risk of bladder cancer. World journal of surgical oncology. 2015;13:297.
6. Sakano S, Berggren P, Kumar R, Steineck G, Adolfsson J, Onelov E, Hemminki K, Larsson P. Clinical course of bladder neoplasms and single nucleotide polymorphisms in the CDKN2A gene. International journal of cancer. 2003;104(1):98-103.
7. Basturk B, Yavascaoglu I, Oral B, Goral G, Oktay B. Cytokine gene polymorphisms can alter the effect of Bacillus Calmette-Guerin (BCG) immunotherapy. Cytokine. 2006;35(1-2):1-5.
8. Guirado M, Gil H, Saenz-Lopez P, Reinboth J, Garrido F, Cozar JM, Ruiz-Cabello F, Carretero R. Association between C13ORF31, NOD2, RIPK2 and TLR10 polymorphisms and urothelial bladder cancer. Human immunology. 2012;73(6):668-72.
9. Wei H, Kamat A, Chen M, Ke HL, Chang DW, Yin J, Grossman HB, Dinney CP, Wu X. Association of polymorphisms in oxidative stress genes with clinical outcomes for bladder cancer treated with Bacillus Calmette-Guerin. PLoS ONE. 2012;7(6):e38533.
10. Ke HL, Chen M, Ye Y, Hildebrandt MA, Wu WJ, Wei H, Huang M, Chang DW, Dinney CP, Wu X. Genetic variations in micro-RNA biogenesis genes and clinical outcomes in non-muscle-invasive bladder cancer. Carcinogenesis. 2013;34(5):1006-11.
11. Xu ZC, Cai HZ, Li X, Xu WZ, Xu T, Yu B, Zou Q, Xu L. ERCC1 C118T polymorphism has predictive value for platinum-based chemotherapy in patients with late-stage bladder cancer. Genetics and molecular research : GMR. 2016;15(2).
12. Hess J, Stelmach P, Eisenhardt A, Rubben H, Reis H, Schmid KW, Bachmann HS. Impact of BCL2 polymorphisms on survival in transitional cell carcinoma of the bladder. Journal of cancer research and clinical oncology. 2017;143(9):1659-70.
