# Supplementary material for: External Replication of Urinary Bladder Cancer Prognostic Polymorphisms in the UK Biobank
Source: Front Oncol. 2019 Oct 18;9:1082. doi: 10.3389/fonc.2019.01082 (PMC6813571; doi:10.3389/fonc.2019.01082)
Supplement: Supplementary file 5 [file Data_Sheet_5.PDF]

Supplementary Table 5. Minor allele frequencies and imputation scores (info) for tested SNPs.

Outcome	SNP	Position (BP)	Allele 1	Allele 2	MAF	Minor Allele	Info score
Age	rs1052133	9798773	C	G	0.23	G	1
	rs710521	189645933	T	C	0.27	C	1
	rs798766	1734239	T	C	0.19	T	1
	rs884225	55274084	T	C	0.10	C	1
	rs1057868	75615006	C	T	0.28	T	1
	rs41515546	125998959	T	C	0.15	C	0.99774
	rs17149636	126018952	A	G	0.15	G	0.99804
	rs17149628	126006965	C	T	0.15	T	0.997627
	rs12666814	125979540	C	T	0.14	T	0.993985
	rs73223045	125992106	G	C	0.15	C	0.99768
	rs12673089	126006133	C	T	0.15	T	0.996984
	rs17149580	125978216	A	G	0.15	G	0.991705
	rs17149630	126006996	C	T	0.15	T	0.997626
	rs7003908	48770702	C	A	0.33	C	0.824001
	rs710886	128026860	C	T	0.37	T	0.998081
	rs9642880	128718068	G	T	0.46	T	1
	rs9344	69462910	G	A	0.44	A	1
	rs217727	2016908	G	A	0.18	A	0.974512
	rs874945	54355451	C	T	0.34	T	0.993033
	rs25487	44055726	T	C	0.36	T	1
Recurrence	rs4645978	15852034	C	T	0.47	C	0.999525
	rs197412	112308953	T	C	0.40	C	1
	rs511918	182579602	G	T	0.41	G	0.992327
	rs1323291	192548543	T	G	0.09	G	0.993577
	rs3795617	192603690	C	T	0.47	T	0.991678
	rs16829458	192768324	G	A	0.07	A	1
	rs11685068	121541012	C	T	0.06	T	0.9919
	rs17235409	219259732	G	A	0.02	A	1
	rs1801282	12393125	C	G	0.12	G	1
	rs1050450	49394834	G	A	0.30	A	0.99929
	rs1052133	9798773	C	G	0.23	G	1
	rs2228001	14187449	G	T	0.40	G	0.995546
	rs1799864	46399208	G	A	0.078308	A	1
	rs4073	74606024	A	T	0.46	A	1
	rs798766	1734239	T	C	0.19	T	1
	rs2910164	159912418	C	G	0.24	C	0.999062
	rs12186785	31410608	T	C	0.09	C	1
	rs4957014	288014	T	G	0.24	T	0.997295
	rs2042329	64067752	T	G	0.41	T	1
	rs2292016	38845860	G	T	0.02	T	0.976753
	rs2278329	38921788	G	A	0.01	A	1
	rs1799964	31542308	T	C	0.21	C	1
	rs2275913	52051033	G	A	0.35	A	0.968288
	rs1800795	22766645	C	G	0.41	C	1
	rs2070744	150690079	C	T	0.38	C	0.987305
	rs1799983	150696111	T	G	0.33	T	0.983157
	rs6463089	42152856	G	A	0.10	A	0.995015
	rs3801192	42161527	C	T	0.09	T	1
	rs1233560	155593438	G	A	0.47	G	0.845252
	rs7003908	48770702	C	A	0.33	C	0.824001
	rs804267	11629241	G	A	0.32	G	0.991159

	rs8191604	11636884	T	G	0.26	G	0.988574
	rs804256	11636862	T	C	0.35	C	0.99245
	rs804276	11625008	G	A	0.42	A	0.990034
	rs4639	11644751	A	G	0.44	G	0.992205
	rs13278062	23082971	G	T	0.50	G	1
	rs2228526	50678717	T	C	0.20	C	1
	rs11199005	121296029	G	A	0.09	A	1
	rs4925	106022789	C	A	0.30	A	1
	rs2505568	36811336	T	A	0.42	T	0.98455
	rs187238	112034988	C	G	0.27	G	0.994289
	rs1695	67352689	A	G	0.35	G	1
	rs2279744	69202580	T	G	0.35	G	0.99522
	rs5742714	102789852	C	G	0.09	G	0.994077
	rs2238151	112211833	T	C	0.35	C	1
	rs4758680	122655352	T	G	0.33	T	0.996788
	rs1544410	48239835	C	T	0.40	T	1
	rs2430561	68552522	T	A	0.114901	A	0.612221
	rs3138056	35868514	C	T	0.32	T	0.980789
	rs2169830	39872056	T	C	0.30	C	0.991923
	rs744154	14015081	G	C	0.28	C	0.996449
	rs243865	55511806	C	T	0.25	T	1
	rs16260	68771034	C	A	0.28	A	0.996119
	rs1042522	7579472	G	C	0.26	G	1
	rs9904341	76210367	G	C	0.31	C	0.992725
	rs2854509	44074597	T	G	0.22	T	0.990178
	rs3213255	44077507	G	A	0.42	G	0.991363
	rs915927	44057227	T	C	0.44	C	0.999689
	rs2854501	44060001	A	G	0.24	A	1
	rs1799782	44057574	G	A	0.06	A	1
	rs25487	44055726	T	C	0.36	T	1
	rs1799793	45867259	C	T	0.33	T	1
	rs5498	10395683	A	G	0.42	G	1
	rs3746162	1114119	C	T	0.22	T	0.998325
	rs7265992	33525407	G	A	0.18	A	0.976046
	rs6060124	33536897	C	A	0.31	A	0.992465
	rs7260770	33552653	G	A	0.33	A	0.996424
	rs4911455	33553062	A	C	0.33	C	0.997023
	rs2173962	33022020	T	C	0.04	C	0.98769
Death	rs1801133	11856378	G	A	0.33	A	1
	rs1395960	163115522	C	T	0.12	T	0.99613
	rs2854461	226011644	C	A	0.35	A	0.994428
	rs2344673	163118052	G	A	0.14	A	1
	rs10917690	163142168	A	G	0.30	G	0.921387
	rs1890398	192771127	C	T	0.35	T	0.997693
	rs12035879	163142555	G	A	0.40	A	1
	rs10753605	163145390	T	C	0.30	C	0.974848
	rs1126579	219000734	T	C	0.47	T	0.999164
	rs1042640	234681544	G	C	0.20	G	1
	rs9282638	119263770	T	C	0.15	C	0.997356
	rs4129009	38774889	T	C	0.15	C	1
	rs762861	3442011	G	C	0.25	C	0.979271
	rs2662238	82499307	G	A	0.45	A	0.992607
	rs10515074	67566193	A	G	0.20	G	0.998354
	rs3756712	309096	A	C	0.38	A	0.996364
	rs2292016	38845860	G	T	0.02	T	0.976753

	rs1799983	150696111	T	G	0.33	T	0.983157
	rs1800795	22766645	C	G	0.41	C	1
	rs2227983	55229255	G	A	0.26	A	1
	rs2017000	55242609	A	G	0.28	G	0.996934
	rs2293347	55268916	C	T	0.11	T	0.991363
	rs35402311	53627403	G	A	0.04	A	1
	rs334358	101910613	G	T	0.20	T	0.994179
	rs868	101911656	A	G	0.20	G	0.994126
	rs1051013	116359339	T	C	0.25	T	1
	rs156697	106039185	A	G	0.35	G	1
	rs4925	106022789	C	A	0.30	A	1
	rs528778	8112143	T	C	0.21	T	0.993889
	rs4987059	636433	G	A	0.05	A	1
	rs1805363	94226952	C	T	0.09	T	1
	rs2279744	69202580	T	G	0.35	G	0.99522
	rs5443	6954875	C	T	0.32	T	1
	rs7180135	41024094	G	A	0.43	G	0.998991
	rs9302752	50719103	T	C	0.29	T	1
	rs17855750	28515228	A	C	0.05	C	1
	rs1800067	14029033	G	A	0.08	A	1
	rs1131341	69748869	G	A	0.04	A	1
	rs1042522	7579472	G	C	0.26	G	1
	rs9906827	78665405	C	T	0.45	T	0.997786
	rs1801018	60985879	T	C	0.42	C	1
	rs2279115	60986837	G	T	0.47	G	0.998126
	rs13181	45854919	T	G	0.36	G	1
	rs915927	44057227	T	C	0.44	C	0.999689
	rs762507	44058098	T	C	0.43	T	0.999682
	rs2854501	44060001	A	G	0.24	A	1
	rs2854509	44074597	T	G	0.22	T	0.990178
	rs3213255	44077507	G	A	0.42	G	0.991363
	rs3730050	40770982	T	C	0.29	T	0.997599
	rs11615	45923653	A	G	0.39	G	1
	rs171140	45865002	C	A	0.45	C	0.995683
	rs1994251	30287328	T	G	0.22	G	0.995433
	rs6024840	54956707	A	G	0.25	G	0.980223
Progression	rs10917690	163142168	A	G	0.302568	G	0.921387
	rs10926466	241521721	C	T	0.263583	C	0.980052
	rs11585883	163104742	T	C	0.0587868	C	0.995822
	rs12038803	241522325	A	G	0.250167	A	0.976809
	rs1323291	192548543	T	G	0.0945522	G	0.993577
	rs1800896	206946897	T	C	0.496355	C	0.993185
	rs6678136	163037317	G	A	0.421169	A	0.9972
	rs2243248	132008644	T	G	0.0678691	G	1
	rs2910164	159912418	C	G	0.24027	C	0.999062
	rs4075958	176784512	G	A	0.258766	A	1
	rs1799983	150696111	T	G	0.331691	T	0.983157
	rs2070744	150690079	C	T	0.378021	C	0.987305
	rs3088440	21968159	G	A	0.0931572	A	0.992401
	rs12628	534242	A	G	0.340834	G	1
	rs3890995	109533529	T	C	0.184181	C	0.990296
	rs9302752	50719103	T	C	0.29363	T	1
	rs2297518	26096597	G	A	0.190104	A	1
	rs1801018	60985879	T	C	0.41806	C	1
	rs11615	45923653	A	G	0.386547	G	1

	rs1800470	41858921	G	A	0.375027	G	0.997955
	rs1800471	41858876	C	G	0.077398	G	1
	rs2073778	20074575	C	T	0.127275	T	0.995009
	rs720012	20098582	G	A	0.127252	A	0.99538

MAF-Minor Allele Frequency; SNP-Single Nucleotide Polymorphism.
